# Specific Functional Features of the Cell Integrity MAP Kinase Pathway in the Dimorphic Fission Yeast *Schizosaccharomyces japonicus*

**DOI:** 10.3390/jof7060482

**Published:** 2021-06-14

**Authors:** Elisa Gómez-Gil, Alejandro Franco, Beatriz Vázquez-Marín, Francisco Prieto-Ruiz, Armando Pérez-Díaz, Jero Vicente-Soler, Marisa Madrid, Teresa Soto, José Cansado

**Affiliations:** Yeast Physiology Group, Department of Genetics and Microbiology, Campus de Excelencia Internacional de Ambito Regional (CEIR) Campus Mare Nostrum, Universidad de Murcia, 30071 Murcia, Spain; elisa.gomez2@um.es (E.G.-G.); afranco@um.es (A.F.); beatriz.vazquez@um.es (B.V.-M.); francisco.prieto@um.es (F.P.-R.); armandojesus.perez@um.es (A.P.-D.); jerovic@um.es (J.V.-S.); marisa@um.es (M.M.)

**Keywords:** fission yeast, MAPK, cell integrity pathway, *S. japonicus*, *S. pombe*, protein kinase C, Pmk1, dimorphism, hyphae

## Abstract

Mitogen activated protein kinase (MAPK) signaling pathways execute essential functions in eukaryotic organisms by transducing extracellular stimuli into adaptive cellular responses. In the fission yeast model *Schizosaccharomyces pombe* the cell integrity pathway (CIP) and its core effector, MAPK Pmk1, play a key role during regulation of cell integrity, cytokinesis, and ionic homeostasis. *Schizosaccharomyces japonicus*, another fission yeast species, shows remarkable differences with respect to *S. pombe*, including a robust yeast to hyphae dimorphism in response to environmental changes. We show that the CIP MAPK module architecture and its upstream regulators, PKC orthologs Pck1 and Pck2, are conserved in both fission yeast species. However, some of *S. pombe*’s CIP-related functions, such as cytokinetic control and response to glucose availability, are regulated differently in *S. japonicus.* Moreover, Pck1 and Pck2 antagonistically regulate *S. japonicus* hyphal differentiation through fine-tuning of Pmk1 activity. Chimeric MAPK-swapping experiments revealed that *S. japonicus* Pmk1 is fully functional in *S. pombe*, whereas *S. pombe* Pmk1 shows a limited ability to execute CIP functions and promote *S. japonicus* mycelial development. Our findings also suggest that a modified N-lobe domain secondary structure within *S. japonicus* Pmk1 has a major influence on the CIP signaling features of this evolutionarily diverged fission yeast.

## 1. Introduction

The evolutionarily conserved MAPK signaling pathways orchestrate multiple and fundamental cellular functions in eukaryotic organisms that assure cell proliferation and survival during unperturbed conditions and in response to environmental stimuli. The intensely studied fission yeast model *Schizosaccharomyces pombe* has three MAPK cascades, known as the stress-activated protein kinase (SAPK) pathway, the cell integrity pathway (CIP), and the pheromone sexual differentiation pathway [1]. Specifically, the CIP regulates a wide range of processes in *S. pombe*, including cell wall composition and integrity, vacuole fusion, cytokinesis, and ionic homeostasis [1,2,3,4]. The core effector of the CIP is MAPK Pmk1, an ERK1/2 ortholog that is the downstream target of a MAPK module composed by MAPKKK Mkh1 and MAPKK Pek1 [1,5]. *S. pombe* Pmk1 becomes activated in a cell-cycle-dependent manner during cytokinesis [6], during nutritional changes such as glucose fasting [3], or in response to environmental insults including heat, cell wall damage, osmotic-saline, and oxidative stresses [5,6]. The architecture of the CIP upstream of the Mkh1–Pek1–Pmk1 MAPK module includes two redundant protein kinase C (PKC) orthologs named Pck1 and Pck2 [6,7,8,9,10,11]. Pck1 and Pck2 share overlapping roles in cell viability and partially complement each other, and their simultaneous deletion elicits a synthetic lethal phenotype [1]. Pck2 operates downstream of the Rho GTPases Rho1 and Rho2, and is the major positive regulator of CIP activity during unperturbed growth and stress, whereas a Rho1–Pck1 branch contributes scarcely to Pmk1 activation during vegetative growth and in response to cell wall stress [6,7,8,9,10,11,12]. A complex cross-regulatory relationship between the CIP and TOR signaling that involves the AKT (Gad8)-dependent translation of both Pck1 and Pck2, allows activation of the CIP during growth and stress [13]. However, Rho1 and Rho2 can also synergistically regulate the biosynthesis of the two major *S. pombe* cell wall polymers α- and β-D-glucan through Pck1 and Pck2 and independently of CIP signaling [1]. The CIP and SAPK pathways functionally interact In Vivo, and share common targets including the transcription factor Atf1 and the RNA binding protein Rnc1 [14].

*S. japonicus*, another fission yeast species, is becoming an emerging model organism to explore developmental and physiological evolutionary changes within the genus *Schizosaccharomyces* thanks to its distinctive features with respect to *S. pombe*. Indeed, *S. japonicus* cells display unusual membrane lipid composition [15], and divide through semi-open mitosis, as opposed to the typical closed mitosis of *S. pombe* cells [16]. In addition, *S. japonicus* utilizes glucose exclusively via fermentation and is essentially respiration defective, whereas *S. pombe* is a respiro-fermentative organism [17]. Most importantly, and unlike the remaining species of the genus, *S. japonicus* yeast cells have the ability to undergo a vigorous and reversible dimorphic switch to hyphae in response to environmental stimuli. Known inducers of hyphal differentiation in this organism include changes in the composition of the growth medium, and DNA damage triggered with camptothecin (CPT), a specific topoisomerase I inhibitor [18,19,20,21].

The *S. japonicus* proteome carries gene orthologs encoding the main components of the three MAP kinase pathways present in *S. pombe* [22]. We have recently described that the stress-responsive functions of the SAPK pathway in *S. pombe* are largely conserved in *S. japonicus*, and include regulation of G2/M progression during the cell cycle, cellular adaptation to multiple stress conditions, and the positive control of chronological lifespan and sexual differentiation [23]. Notably, the SAPK pathway acts in concert with a quorum sensing mechanism mediated by aromatic alcohols that represses *S. japonicus* yeast to hypha differentiation in response to increased population density [23]. In contrary, *S. japonicus* mutants lacking the putative CIP MAP kinase module components Mkh1, Pek1, and MAPK Pmk1 display defective mycelia formation in solid medium [22], suggesting that this signaling pathway may play instead a positive role to modulate yeast to hypha dimorphic transition. However, the precise biological functions of the *S. japonicus* CIP during growth, stress, and mycelial differentiation, together with the possible role of PKC orthologs Pck1 and Pck2 as upstream regulators of this signaling cascade, are currently unknown. In this work we show that, despite the existence of some degree of functional conservation, certain biological features of CIP signaling have evolved separately in *S. japonicus* and *S. pombe*, and that Pck1 and Pck2 modulate Pmk1 MAPK activity during *S. japonicus* dimorphic switch in an opposite fashion. Finally, structural changes at the N-lobe of *S. japonicus* Pmk1 likely played a significant role in modulating CIP signaling to cope with the specific developmental requirements of this fission yeast species.

## 2. Materials and Methods

### 2.1. Strains, Growth Conditions, and Reagents

References and genotypes of *S. pombe* and S. *japonicus* strains employed in this work are listed in Table S1. They were grown with shaking at 30 °C in yeast extract plus supplements (YES) or Edinburgh minimal medium (EMM2) with 2% glucose, and supplemented with adenine, leucine, histidine, or uracil (100 mg/L, Sigma-Aldrich, St. Louis, MO, USA) [24]. Mutant strains with single/multiple gene deletions, or expressing genomic fusions, were obtained by transformation, or after tetrad or random spore dissection and analysis of appropriate crosses in sporulation medium (SPA) [25]. In stress treatments, log-phase cultures (OD_600_ = 0.5; ~10^6^ cells/mL) were supplemented with either KCl (osmotic-saline stress; Sigma-Aldrich), caspofungin (cell wall stress; Sigma-Aldrich), or hydrogen peroxide (oxidative stress; Sigma-Aldrich). In glucose starvation experiments, cells grown in YES medium with 7% glucose were recovered by filtration, and resuspended in the same medium lacking glucose and osmotically equilibrated with 3% glycerol. In yeast to hyphae induction experiments with *S. japonicus*, log-phase cultures grown in YES medium with 6% glucose were treated with camptothecin (CPT; Sigma-Aldrich) to a final concentration of 0.2 μM. YEMA [18] and RGE [26] media supplemented with 2% agar were used to quantify *S. japonicus* mycelial growth. *S. japonicus* transformation by electroporation was performed exactly as described [27].

### 2.2. Gene Disruption and Gene Fusion

Sequences of *S. japonicus* genes were obtained from the annotated database at *EnsemblFungi* (http://fungi.ensembl.org/Schizosaccharomyces_japonicus/Info/Index?db=core (accessed on 14 June 2021)). The *S. japonicus pck1+, pck2+,* and *pmk1+* null mutants were obtained by entire deletion of the corresponding coding sequences by PCR-mediated strategy using plasmids pFK14 (*S. japonicus ura4+* gene cloned into pGEMT-easy vector; [28]) or pFA6a-natMX6 [29] as templates, and their replacement with either *ura4+* or *natMX6* cassettes flanked by long 5′ and 3′UTRs of respective genes following a PCR approach [30]. Oligonucleotides employed to obtain each one of the transformation cassettes are shown in Table S2.

To construct the integrative plasmid pFA6a-Pmk1^Sj^:GFP-*natMX6*, the *pmk1^+Sj^* ORF plus 5′ regulatory sequences were amplified by PCR using *S. japonicus* genomic DNA as a template, the 5’-oligonucleotide Pmk1Sj:GFP-*Sal*I-5′, which hybridizes −309 to −288 bp upstream of the *pmk1*^+Sj^ start codon and contains a *Sal*I site, and the 3′-oligonucleotide Pmk1Sj:GFP-*Pac*I-3’, which hybridizes at the 3′ end of *pmk1*^+*Sj*^ ORF before the stop codon, and contains a *Pac*I site. The obtained DNA fragment was cloned into plasmid pFA6a-GFP:*natMX6* [29] digested with *Sal*I and *Pac*I. Plasmid pFA6a-Pmk1^Sj^:GFP-*natMX6* was then digested at the unique *Swa*I site within *pmk1^+Sj^* ORF, and transformed into strain NIG2028. *Nat^R^* transformants were obtained, and the correct integration and expression of the fusion was verified by DNA sequencing and Western blot analysis.

### 2.3. Construction of S. pombe and S. japonicus Strains Expressing Wild-Type and Chimeric Pmk1–HA Fusions

To obtain the integrative plasmid pSL-Pmk1^Sj^:HA, the *pmk1^+Sj^* ORF plus 5′ regulatory sequences were amplified by PCR using *S. japonicus* genomic DNA as a template, the 5′-oligonucleotide PromPmk1Jp-*Not*I-5′, which hybridizes at positions −498 to −475 bp upstream of the *pmk1*^+^ start codon and contains a *Not*I site, and the 3′-oligonucleotide Pmk1Jp:HA-*BamH*I-3′, which hybridizes at the 3′ end of *pmk1*^+*Sj*^ ORF and incorporates an additional 33-nucleotide sequence encoding one HA epitope (sequence -GYPYDVPDYAG), and contains a *BamH*I site. The obtained PCR fragment was digested with *Not*I and *BamH*I and cloned into plasmid pSL, which is a derivative of plasmid pS0550 (kindly provided by S. Oliferenko) that incorporates the *nmt1^+^* 3′ regulatory sequences. To obtain integrative plasmid pSL-Pmk1^Sj^(ins 82-105)–HA, we followed a modular PCR-based approach employing *S. japonicus* genomic DNA and different nucleotide pairs. A first DNA fragment was amplified with the oligonucleotide Pmk1Jp-EXON-5’, which hybridizes 620 to 691 bp downstream of *pmk1*^+*Sp*^ ORF and incorporates a 71-nucleotide sequence encoding the 24 aa motif present in Pmk1^Sp^ N-lobe, and 3′-oligonucleotide Pmk1Jp:HA-*BamH*I-3′. A second DNA fragment was amplified with 5′-oligonucleotide PromPmk1Jp-*Not*I-5′ and 3′-oligonucleotide Pmk1Jp-EXON-3’, which hybridizes 424 to 448 bp downstream of *pmk1*^+*Sj*^ ORF plus and incorporates a reverse complement 71-nucleotide sequence from Pmk1^Sp^ ORF described above. Both DNA fragments were gel purified and used as templates in a second-round PCR in the presence of PromPmk1Jp-*Not*I-5′ and Pmk1Jp:HA-*BamH*I-3′. The resulting PCR fragment was digested with *Not*I/*BamH*I and cloned into plasmid pSL. To obtain integrative plasmid pSL-Pmk1^sp^:HA6H, the *pmk1*^+Sp^ ORF plus regulatory sequences were amplified by PCR using genomic DNA from *S. pombe* strain MM2 as template [5], and 5′-oligonucleotide PromPmk1Sp-*Xba*I-5′, which hybridizes at positions 330 to 313 upstream of the *pmk1*^+Sp^ start codon and contains a *Xba*I site, and the 3′-oligonucleotide Pmk1Sp–HA-3′, which hybridizes at the 3′ end of *pmk1*^+Sp^ ORF and incorporates a 48-nucleotide sequence encoding one HA epitope plus six consecutive histidine residues, and a *Sma*I site. The DNA fragment was digested with *Xba*I and *Sma*I and cloned into plasmid pSL. For plasmid pSL-Pmk1^Sp^ (Δ82-105):HA, the *pmk1*^+Sp^ ORF plus regulatory sequences were amplified in by PCR using *S. pombe* genomic DNA as template, the 5′-oligonucleotide PromPmk1Sp-*Xba*I-5′, and 3′-oligonucleotide Pmk1Sp-ΔExon-3′, which hybridizes −326 to −346 bp upstream and 774 to 792 bp downstream of *pmk1*^+*Sp*^ ORF. A second DNA fragment was amplified with the 5′-oligonucleotide Pmk1Sp-ΔExon-5′, which is complementary and the reverse of Pmk1Sp-ΔExon-3′, and 3′-oligonucleotide Pmk1Sp–HA-3′. Both fragments were gel purified and used in a second-round PCR as templates in the presence of the external oligonucleotides PromPmk1Sp-*Xba*I-5′ and Pmk1Sp–HA-3′. The resulting PCR fragment was digested with *Xba*I and *Sma*I and cloned into plasmid pSL. All of the above plasmids were digested at the unique *Afe*I site within *ura4^+Sj^*, and the linear fragments were transformed into the *S. japonicus pmk1^+^::NatR* strain EGJ83. *ura4^+^* transformants were obtained, and the correct integration and expression of the HA fusions was verified by sequencing and Western blot analysis.

To express the chimeric constructs described above in *S. pombe*, the respective Pmk1–HA fused cassettes plus 5′ regulatory sequences were gel purified from plasmids pSL-Pmk1^Sj^:HA, pSL-Pmk1^Sj^(ins 82-105)–HA, pSL-Pmk1^Sp^:HA, and pSL-Pmk1^Sp^(Δ82-105):HA after digestion with *Xba*I and *Sma*I, and cloned into *S. pombe* integrative plasmid pJK148. The resulting plasmid constructs were digested at the unique *Nru*I site within *Leu1^+Sp^*, and the linear fragments were transformed into the *S. pombe pmk1^+^::NatR* strain MM1904. *Leu1^+^* transformants were obtained, and the correct integration and expression of the fusions was verified by sequencing and Western blot analysis.

### 2.4. Detection and Quantification of Activated and Total Pmk1 Levels

Extracts from *S. pombe* and *S. japonicus* cells collected during unperturbed growth, or after stress or CPT treatments were prepared under native conditions employing chilled acid-washed glass beads and lysis buffer (10% glycerol, 50 mM Tris/HCl pH 7.5, 15 mM Imidazole, 150 mM NaCl, 0.1% Nonidet NP-40, plus specific protease and phosphatase inhibitor, Sigma-Aldrich), as described [31]. Dually phosphorylated Pmk1 was detected employing a rabbit polyclonal anti-phospho-p44/42antibody (9191L; Cell Signaling). Total Pmk1 levels were detected with either mouse monoclonal anti-HA (Pmk1–HA fusions; clone 12CA5; Roche Molecular Biochemicals, Basel, Switzerland), or anti-GFP antibody (Pmk1–GFP fusions; Roche Molecular Biochemicals). Rabbit polyclonal anti-Cdk1/Cdc2 antibody (PSTAIR; Millipore) was used for loading control. Immunoreactive bands were revealed with anti-mouse or anti-rabbit peroxidase-conjugated secondary antibodies (both from Sigma-Aldrich), and the ECL system (GE-Healthcare, Uppsala, Sweden). Densitometric quantification of Western blot signals from 16-bit .jpg digital images of blots was performed using ImageJ [32]. Relative units for Pmk1 activation were estimated by determining the signal ratio of the anti-phospho-p44/42 blot (activated Pmk1) with respect to the anti-HA or anti-GFP blot (total Pmk1) at each time point. Unless otherwise stated, results shown correspond to experiments performed as biological duplicates or triplicates. Mean relative units ± SD and/or representative results are shown. *p*-values were analyzed by unpaired Student’s t-test.

### 2.5. Co-immunoprecipitation

Whole-cell extracts (5 mg total protein) were prepared in lysis buffer (50 mM Tris/HCl pH 7.5, 0.5% sodium deoxicholate, 150 mM NaCl, 1% NP-40 and protease inhibitor (Sigma-Aldrich), and incubated with anti-HA monoclonal antibody (12CA5; Roche Molecular Biochemicals) and protein A sepharose beads for 4 h at 4 °C. The beads were washed two times with lysis buffer, two times with washing buffer 2 (50 mM Tris/HCl pH 7.5, 0.05% sodium deoxicholate, 500 mM NaCl, 0.1% NP-40), one time with washing buffer 3 (50 mM Tris/HCl pH 7.5, 0.05% sodium deoxicholate, 0.1% NP-40), and resuspended in sample buffer. Proteins were separated by SDS-PAGE, transferred to nitrocellulose filters (GE Healthcare), and hybridized with either mouse monoclonal anti-HA antibody (Pmk1–HA fusions; clone 12CA5; Roche Molecular Biochemicals), or anti-GFP antibody (Pek1–GFP fusions; Roche Molecular Biochemicals), followed by immunodetection with anti-mouse peroxidase-conjugated secondary antibody (Sigma-Aldrich), and the ECL system (GE Healthcare).

### 2.6. Plate Assay of Stress Sensitivity for Growth

*S. pombe* and *S. japonicus* wild-type and mutant strains were grown in YES liquid medium to OD_600_ = 0.5, and appropriate decimal dilutions were spotted per duplicate on YES solid medium or in the same medium supplemented with varying concentrations of caspofungin (Sigma-Aldrich), and magnesium chloride plus the calcineurin inhibitor FK506 (Tracholimus; Sigma-Aldrich). Plates were incubated at 30 °C for 3 days and then photographed. All the assays were repeated at least three times with similar results. Representative experiments are shown in the corresponding figures.

### 2.7. Quantification of Mycelial Growth during Nutritional Stress

Approximately 2.10^6^ cells from log-phase cultures (OD_600_= 0.5) of wild-type and mutant strains growing in YES medium were spotted on YEMA or RGE plates, incubated at 30 °C for 7 days, and then photographed and saved as 16-bit .jpg digital images. The area of mycelial expansion was outlined for each strain (*n* ≥ 6) by freehand, and measured with ImageJ using the *analyze tool* [32].

### 2.8. Microscopy Observation

**Figure 1:**
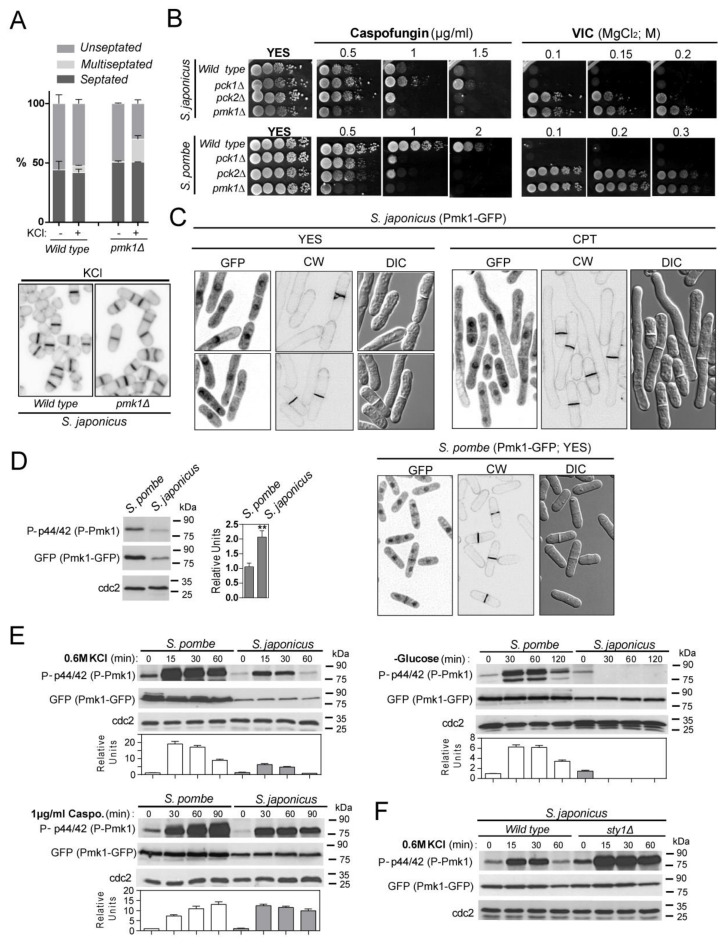
Functional roles, localization, and activation pattern of the CIP MAPK Pmk1 in *S. japonicus.* (**A**) *Upper*: Exponentially growing *S. japonicus* wild-type and *pmk1*Δ strains were incubated for 10 h in YES medium plus 0.6M KCl, and the percentage of unseptated, septated, and multiseptated cells (represented as mean ± SD from biological triplicates; number of total cells ≥ 200) was determined by fluorescence microscopy after calcofluor white staining. *Lower*: Representative images are shown; (**B**) Decimal dilutions of the indicated strains growing in YES medium were spotted onto YES plates supplemented with caspofungin (0.5–2 µg/mL) (upper panels), and 0.5 µg/mL FK506 plus 0.1, 0.15, or 0.2 M MgCl_2_ (VIC assay; lower panels), and incubated for 3 days at 30 °C before being photographed. Representative experiments are shown; (**C**) *S. japonicus* and *S. pombe* wild-type cells expressing genomic Pmk1–GFP fusions were grown in YES medium to mid-log phase or treated for 6 h with 0.2 μM CPT (*S. japonicus*), and observed both by fluorescence and DIC microscopy after calcofluor white staining. Images were acquired as single medial plane images and are inverted for fluorescence; (**D**) *S. pombe* and *S. japonicus* wild-type strains expressing genomic Pmk1–GFP fusions were grown in YES medium to mid-log phase, and activated/total Pmk1 were detected with anti-phospho-p44/42 and anti-GFP antibodies, respectively. Anti-Cdc2 was used as a loading control. Relative units as mean ± SD (biological triplicates) for Pmk1 phosphorylation (anti-phospho-p44/42 blot) were determined with respect to the internal control (anti-GFP blot). **, *p* < 0.005; as calculated by unpaired Student’s *t*-test; (**E**) *S. pombe* and *S. japonicus* wild-type strains expressing genomic Pmk1–GFP fusions were grown in YES medium to mid-log phase, and treated with 0.6 M KCl, 1 µg/mL caspofungin, or recovered by filtration and resuspended in osmotically equilibrated medium lacking glucose for the indicated times. Activated/total Pmk1 was detected with anti-phospho-p44/42 and anti-GFP antibodies, respectively. Anti-Cdc2 was used as a loading control. Relative Pmk1 activity units as mean ± SD (biological duplicates) were determined as described in (**C**); (**F**) *S. japonicus* wild-type and *sty1*Δ strains expressing a genomic Pmk1–GFP fusion were grown in YES medium to mid-log phase, and treated with 0.6 M KCl for the indicated times. Activated/total Pmk1 were detected with anti-phospho-p44/42 and anti-GFP antibodies, respectively. Relative Pmk1 activity units as mean ± SD (biological duplicates) were determined as above.

**Figure 2:**
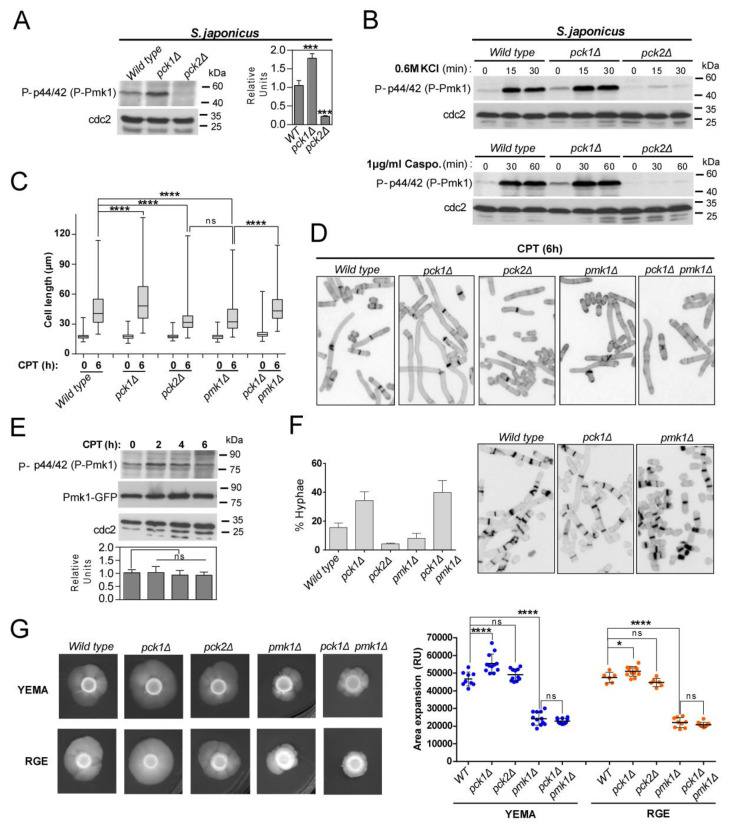
Antagonistic control of Pmk1 activity by PKC orthologs Pck1 and Pck2 modulates *S. japonicus* hyphal differentiation. (**A**) *S. japonicus* strains of the indicated genotypes were grown in YES medium to mid-log phase, and activated Pmk1 were detected with anti-phospho-p44/42, whereas anti-Cdc2 was used as a loading control. Relative units as mean ± SD (biological triplicates) for Pmk1 phosphorylation (anti-phospho-p44/42 blot) were determined with respect to the internal control (anti-Cdc2 blot). ***, *p* < 0.001; as calculated by unpaired Student’s *t*-test; (**B**) *S. japonicus* strains of the indicated genotypes were grown in YES medium to mid-log phase, treated with either 0.6 M KCl (upper panels) or 1 µg/mL caspofungin (lower panels) for the indicated times, and activated Pmk1 was detected with anti-phospho-p44/42, whereas anti-Cdc2 was used as a loading control. Results from representative experiments are shown; (**C**) Exponentially growing *S. japonicus* cells of the indicated genotypes were inoculated at an initial cell density of 10^6^ cells/mL in YES medium with 6% glucose and incubated for 0 and 6 h with 0.2 μM CPT. Cell length at each time point is represented as box and whisker plots. Data obtained after quantification of one experiment performed per triplicate (*n* ≥ 400 cells/strain) is shown. ****, *p* < 0.0001; ns, not significant, as calculated by one-way ANOVA; (**D**) Representative images of strains analyzed in (C) were obtained by fluorescence microscopy after calcofluor white staining; (**E**) Exponentially growing *S. japonicus* wild-type cells (YES medium with 6% glucose) expressing a Pmk1–GFP fusion were treated with 0.2 μM CPT for the indicated times. Activated/total Pmk1 were detected with anti-phospho-p44/42 and anti-GFP antibodies, respectively. Anti-Cdc2 was used as a loading control. Relative units as mean ± SD (biological duplicates) for Pmk1 phosphorylation (anti-phospho-p44/42 blot) were determined with respect to the internal control (anti-GFP blot). ns, not significant, as calculated by unpaired Student’s *t*-test; (**F**) *Left*: *S. japonicus* strains of the indicated genotypes were grown in YES medium plus 0.2 μM CPT for 12 h, and the percentage of hyphae were quantified. Percentages are expressed as mean ± SD and correspond to biological duplicates (*n* ≥ 200 cells/sample). *Right*: Representative images of strains were obtained by fluorescence microscopy after calcofluor white staining; (**G**) *Left*: cells from log-phase cultures of the indicated strains growing in YES medium (~2.10^6^ cells in each case) were spotted on YEMA and RGE plates, incubated at 30 °C for 7 days, and then photographed. *Right*: the total area of mycelial expansion (expressed as relative units, RU) was measured for each strain genotype (*n* ≥ 6) and is represented as scatter plot. *, *p* < 0.05; ****, *p* < 0.0001; ns, not significant, as calculated by one-way ANOVA.

Fluorescence images in Figure 1C were acquired using a Leica Thunder imager by either fluorescence or DIC microscopy as single medial plane images from a set of 20 stacks (0.3 µm each), and processed through ImageJ [32]. Fluorescence images in Figure 1A, Figure 2D,E and Figure 4E were obtained with a Leica DM4000B microscope and processed using IM500 Image Manager software. Calcofluor white was employed for cell wall/septum staining as described [24]. To quantify the increase in cell length during hyphal induction experiments with CPT, samples were taken at the indicated times and fixed immediately with formaldehyde [33]. After staining with calcofluor white the length of mononuclear late G2 cells was measured. Three biological replicates (*n* ≥ 400) were scored for each strain genotype.

## 3. Results

### 3.1. Environmental Control of S. japonicus Cell Integrity MAPK Pathway by the PKC Orthologs Pck1 and Pck2

To explore the evolutionary conservation of the cell integrity pathway within the fission yeast clade, we obtained a *S. japonicus* strain deleted in MAPK Pmk1 by homologous recombination (see Materials and Methods). *S. pombe pmk1*Δ cells display a multiseptated phenotype when cultured in medium supplemented with sorbitol, calcofluor, KCl, or caffeine [34,35,36]. While the percentage of septated and multiseptated cells was very similar in *S. japonicus* wild-type and *pmk1*Δ cells growing in the presence of sorbitol, calcofluor and caffeine, multiseptation increased significantly in *pmk1*Δ cells treated with KCl (Figure 1A), suggesting that Pmk1 might play a less meaningful role during control of septation in this organism. As compared to the wild-type strain, *S. japonicus pmk1*Δ cells were growth sensitive to the β-glucan synthase inhibitor caspofungin but to a lesser extent than the *S. pombe pmk1*Δ mutant (Figure 1B), indicating that Pmk1 likely regulates cell wall synthesis and/or integrity in both fission yeast species. Calcineurin and Pmk1 play antagonistic roles during control of calcium homeostasis in *S. pombe*, and lack of Pmk1 activity allows cell growth in the presence of MgCl_2_ when calcineurin activity is blocked with the specific inhibitor FK506. This phenotype, known as “VIC” (viable in the presence of immunosuppressant and chloride ion) [37], was also displayed by *S. japonicus pmk1*Δ cells (Figure 1B), revealing that a functional crosstalk between calcineurin and Pmk1 signaling is conserved within the *Schizosaccharomyces* genus.

Pmk1 MAPKs in *S. pombe* and *S. japonicus* share a high amino-acid sequence identity, particularly at the N- and C-lobes (~85% identity within a 336 amino-acid sequence), and decreases gradually towards the C-tail (Figure S2). The glycine-ATP-phosphate-binding loop, the invariant lysine and glutamic acid residues required for full kinase activity, the ERK-type TEY activation loop, and the common docking (CD) domain required for MAPK binding to upstream activators and downstream targets, are conserved in *S. japonicus* Pmk1 with respect to the corresponding *S. pombe* counterpart (Figure S2). However, a striking feature of *S. japonicus* Pmk1 secondary structure is the absence at its N-lobe of a 24 aa motif (sequence –ITCIYDLDIINPYNFNEVYIYEEL-), which encompasses the full subdomain IV and part of subdomain V (hinge region), and includes the putative gatekeeper residue E104 (Figure S2). A global BlastP search of yeast and fungal proteomes indicated that this unusual N-lobe conformation is highly specific of *S. japonicus* Pmk1, as it was found in only three hypothetical cell integrity MAPKs from ascomycetes belonging to order *Chaetothyriales* (Gen Pep: DV737_g786 and DV736_g4926), and the species *Ophiocordyceps camponoti* (Gen Pep: CP532_1836) (Figure S3).

*S. pombe* Pmk1 displays a nucleo-cytoplasmic localization during unperturbed growth and is targeted to the septum during cytokinesis [5]. We found that, in contrast to *S. pombe*, in *S. japonicus* a genomic Pmk1–GFP fusion shows nucleo–cytoplasmic location but it is not targeted to the septum, as confirmed after observation of septum staining with calcofluor white and DIC microscopy of yeast cells growing exponentially in rich medium, or in hyphae produced after treatment with CPT (Figure 1C). Incubation with an anti-phospho-p44/42 ERK antibody, which detects dually phosphorylated Pmk1 in *S. pombe* [5], revealed a single phosphorylation signal with the predicted molecular weight of Pmk1 (~47 Da) in extracts from *S. japonicus* wild-type cells, but not in those from the *pmk1*Δ mutant (Figure S2). We note that the relative basal Pmk1 phosphorylation level was significantly higher (2–2.5 times) in vegetatively growing *S. japonicus* versus *S. pombe* cells expressing genomic Pmk1–GFP fusions (Figure 1D). *S. pombe* Pmk1 becomes activated in response to several stimuli including saline stress, glucose starvation, and cell wall damage induced with caspofungin [3,5,7,12]. Pmk1 also became activated in *S. japonicus* cells treated with salt (KCl) or caspofungin, but to different magnitudes and dynamics than in *S. pombe* (Figure 1E). Remarkably, Pmk1 activity decreased quickly during glucose starvation (Figure 1E), suggesting that the CIP is not operative in *S. japonicus* under this specific stimulus. In *S. pombe*, transcriptional induction of tyrosine and serine/threonine phosphatases by the SAPK pathway through the Sty1–Atf1 branch promotes the dephosphorylation of activated Pmk1 during vegetative growth and in response to stress, thus revealing a cross-inhibitory mechanism between the two MAPK cascades [4]. Indeed, both basal and the induced phosphorylation of Pmk1 under saline stress increased significantly in a *S. japonicus sty1*Δ mutant [23], as compared to wild-type cells (Figure 1F), suggesting that the interplay between the SAPK and CIP pathways is conserved in both *Schizosaccharomyces* species.

The two non-essential PKC orthologs, Pck1 and Pck2 [1,38], are core upstream activators of the CIP during growth and stress in *S. pombe* [6,7]. *S. japonicus* proteome also carries two putative orthologs for Pck1 (SJAG_01787) and Pck2 (SJAG_00610), which share ~52 and ~66% amino-acids sequence identity with the respective *S. pombe* counterparts. Both kinases have the conserved putative HR1 rho-binding repeat domains that in *S. pombe* mediate their binding to Rho GTPases Rho1 and Rho2 [39], plus the C2 (Ca^2+^-binding), Ps (pseudosubstrate), and C1 (diacylglycerol-binding) motifs present in *S. pombe* Pck1 and Pck2 (Figures S4 and S5) [39]. The canonical phosphorylated residues at the kinase domain within the AL (activation loop), TM (Turn motif), and HM (hydrophobic motif), are also conserved (Figures S4 and S5) [39]. *S. japonicus* single mutants lacking either Pck1 or Pck2 were viable; however, we were unable to obtain a double-mutant strain lacking both kinases, suggesting that they partially complement each other and share essential functional roles for cell viability as in *S. pombe* [40,41]. As compared to wild-type cells, Pmk1 basal activity was strongly reduced (>80%) in *S. japonicus pck2*Δ cells during vegetative growth, and increased approximately by two-fold in the *pck1*Δ mutant (Figure 2A). Accordingly, in *pck2*Δ cells reduced Pmk1 activity was accompanied by a less severe VIC phenotype than that of *pmk1*Δ cells (Figure 1B). Thus, *S. japonicus* Pck2 is a major activator of the Pmk1 MAPK module during unperturbed growth. *S. pombe* Pmk1 activation during osmotic–saline stress with KCl is fully dependent on Pck2 function, whereas Pck1 does not play a meaningful role during this response [6]. This signaling mechanism is also conserved within the *S. japonicus* CIP, since Pck2 deletion completely abolished Pmk1 activation in the presence of KCl (Figure 2B). Despite showing very low MAPK levels, the growth sensitivity to caspofungin of *S. japonicus pck2*Δ cells was negligible as compared to that of *pmk1*Δ cells (Figure 1B). Notably, and in contrast to the *S. pombe pck1*Δ mutant, *S. japonicus* cells lacking Pck1 were growth resistant to this compound (Figure 1B). In *S. pombe*, Pck2 is a major factor responsible for Pmk1 activation during cell wall damage induced with caspofungin, while Pck1 likely plays a secondary role [7]. Instead, caspofungin-induced Pmk1 activation in *S. japonicus* was strictly Pck2-dependent, as Pck1 absence did not reduce MAPK activation and/or dynamics (Figure 2B). Together, the above findings suggest that respective positive and negative modulation of CIP activity by Pck2 and Pck1 has an opposite impact on *S. japonicus* cell wall integrity, and that MAPK activation in response to cell wall damage is transduced to the MAPK module exclusively through Pck2.

### 3.2. Fine-Tuning of Pmk1 Activity by Pck1 and Pck2 Modulates Antagonistically S. japonicus Hyphal Differentiation

As compared to wild-type cells, *S. japonicus* null mutants lacking the putative CIP MAP kinase module components Mkh1 (MAPKKK), Pek1 (MAPKK), and Pmk1 (MAPK), show defective mycelia formation when growing in a malt-extract-based solid medium (YEMA) [22], suggesting that the CIP plays a positive role to modulate yeast to hypha dimorphic transition. However, the possible role of the upstream activators of the MAP kinase module Pck1 and Pck2, and the functional relevance of this control, have not been addressed. We note that the average cell length after 6 h of incubation in rich medium supplemented with 0.2 μM CPT, which induces a quick yeast to hyphae transition, was significantly higher in *S. japonicus pck1*Δ cells than in wild-type cells (81.5 + 15.4 μm in *pck1*Δ cells versus 55.1 + 24.9 μm in wild-type cells; Figure 2C,D). On the contrary, CPT-induced differentiation was markedly impaired to a similar extent in both *pck2*Δ and *pmk1*Δ mutants (34.2 + 12.6 and 37.6 + 16.2 μm in *pck2*Δ and *pmk1*Δ cells, respectively; Figure 2C,D). Total and activated basal Pmk1 levels remained unchanged during the early stages of hyphal initiation (Figure 2E). Interestingly, the enhanced hyphal differentiation of *pck1*Δ cells was suppressed in the absence of Pmk1 function only partially (47.2 + 16.2 μm in *pck1*Δ *pmk1*Δ double mutant; Figure 2C,D). In line with the above findings, the *pck1*Δ mutant produced a higher ratio of filaments and/or hypha than the wild-type strain after 12 h of incubation with CPT, whereas this percentage became significantly reduced in *pck2*Δ and *pmk1*Δ mutants, but not in a *pck1*Δ *pmk1*Δ strain (Figure 2F).

The mycelial area of expansion of the *pmk1*Δ mutant, but not that of the *pck2*Δ strain, decreased significantly with respect to the wild type when incubated for six days in YEMA or RGE plates (Figure 2G). In contrary, mycelium production was enhanced in *pck1*Δ cells growing in both inducing media, and this phenotype was fully suppressed by simultaneous deletion of Pmk1 (Figure 2G). Taken together, the above findings suggest that Pmk1 plays a major positive role to control both the early and late phases of hyphal differentiation in *S. japonicus*, whereas the positive impact of Pck2 signaling seems restricted to the initial differentiation step. Conversely, Pck1 negatively modulates this process by a mechanism that fully depends on the reduction of Pmk1 activity during the late stages of mycelium production (plate assays), but not at the early stages of the dimorphic switch (CPT treatment).

### 3.3. Species-Specific N-lobe Domain Architecture Accounts for Differential Control of Pmk1 Function in S. pombe and S. japonicus during Growth, Stress, and Hyphal Differentiation

**Figure 3:**
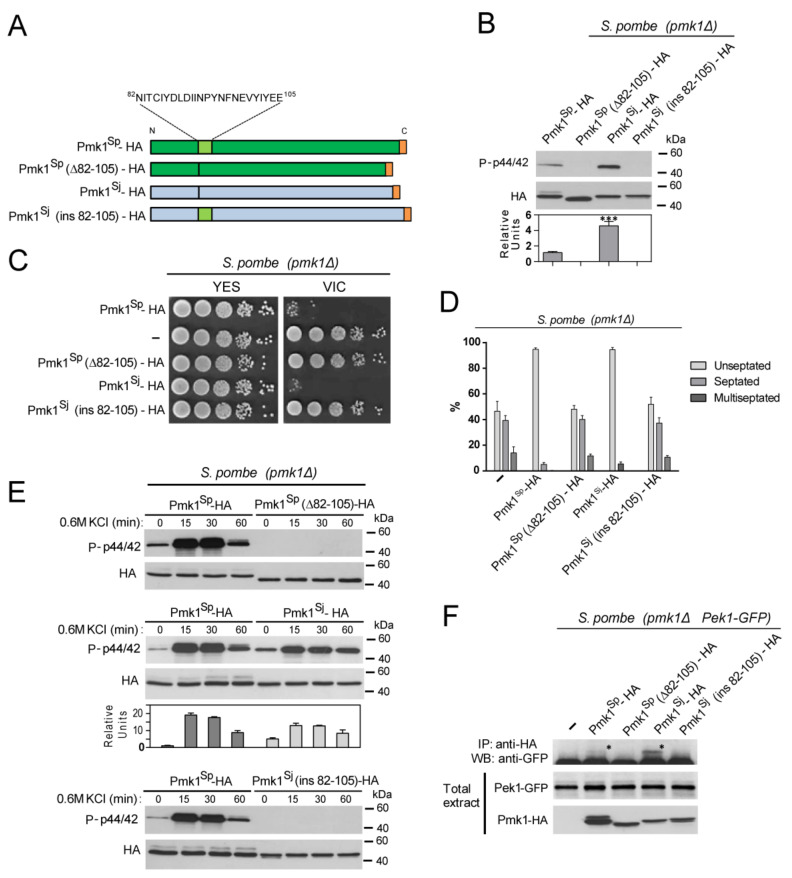
*S japonicus* Pmk1 is fully functional in *S. pombe***.** (**A**) *S. pombe* and *S. japonicus* Pmk1–HA chimeric constructs. Pmk1^Sp^–HA: *S. pombe* wild-type Pmk1 construct showing the N-lobe subdomain IV–V conserved 24 aa motif (light green) is shown. Pmk1^Sp^ (Δ82-105)–HA: *S. pombe* Pmk1 version lacking the conserved N-lobe conserved motif. Pmk1^Sj^–HA: *S. japonicus* wild-type Pmk1 construct that lacks the conserved N-lobe conserved motif. Pmk1^Sj^ (ins82-105)–HA: *S. japonicus* Pmk1 version with an insertion of the *S. pombe* Pmk1 N-lobe 24 aa motif (light green); (**B**) *S. pombe pmk1*Δ cells expressing the Pmk1–HA chimeric constructs described in (**A**) were grown in YES medium to mid-log phase, and activated/total Pmk1 were detected with anti-phospho-p44/42 and anti-HA antibodies, respectively. Relative units as mean ± SD (biological triplicates) for Pmk1 phosphorylation (anti-phospho-p44/42 blot), were determined with respect to the anti-HA blot (total Pmk1). ***, *p* < 0.001; as calculated by unpaired Student’s *t*-test; (**C**) Decimal dilutions of *S. pombe pmk1*Δ cells (-) and those expressing the indicated Pmk1–HA chimeric constructs, were spotted onto YES plates supplemented with 0.5 µg/mL FK506 plus 0.2 M MgCl_2_ (VIC assay), and incubated for 3 days at 28 °C before being photographed. A representative experiment is shown; (**D**) *S. pombe pmk1*Δ cells (-) and those expressing the indicated Pmk1–HA chimeric constructs were grown for 24 h in YES medium plus 1M sorbitol, and the percentage of septated/multiseptated cells (represented as mean ± SD from biological triplicates; number of cells ≥ 200) was determined by fluorescence microscopy after calcofluor white staining; (**E**) *S. pombe* strains expressing the indicated Pmk1–HA chimeric constructs were grown in YES medium to mid-log phase and treated with 0.6 M KCl for the indicated times. Activated/total Pmk1 were detected with anti-phospho-p44/42 and anti-HA antibodies, respectively. Relative units as mean ± SD (biological duplicates) for Pmk1 phosphorylation (anti-phospho-p44/42 blot) were determined with respect to the anti-HA blot (total Pmk1). Either representative results or those obtained from biological triplicates are shown; (**F**) Co-immunoprecipitation of Pmk1–HA chimeric constructs and a Pek1–GFP (MAPKK) genomic fusion from yeast extracts obtained from vegetatively growing cultures from the indicated strains. Results from a representative experiment are shown. IP, immunoprecipitation; WB, Western blot; *, specific Pek1-GFP fusion.

The unusual secondary structure of *S. japonicus* Pmk1 at its N-lobe lacking most parts of subdomains IV and V (Figure S2), together with the results obtained so far, prompted us to thoroughly explore the possible impact of MAPK conformation on the evolutionary conservation of Pmk1 functions in the two fission yeast species. To this end, we constructed *S. pombe* and *S. japonicus pmk1*Δ mutants expressing distinct genomic Pmk1–HA fused chimeras under the control of their respective wild-type promoters (named as Pmk1^Sp^ and Pmk1^Sj^ thereafter) (Figure 3A). In contrast to the wild-type Pmk^Sp^–HA fusion, expression in *S. pombe pmk1*Δ cells of a MAPK version lacking the conserved N-lobe 24 aa motif (Pmk1^Sp^ (Δ82-105)–HA; Figure 3A), did not show detectable phosphorylation during unperturbed growth (Figure 3B), suggesting that this motif is essential for kinase activation and/or activity. Accordingly, Pmk1^Sp^ (Δ82-105)–HA failed to suppress both the VIC and multiseptated phenotypes of *S. pombe pmk1*Δ cells (Figure 3C,D), and was not activated in response to saline stress (Figure 3E). Indeed, the relative basal activity of a *S. japonicus* Pmk1 fusion expressed in *S. pombe* (Pmk1^Sj^–HA; Figure 3A) was significantly higher than that of the endogenous MAPK (Figure 3B). Pmk1^Sj^–HA suppressed the VIC and multiseptated phenotypes of *S. pombe pmk1*Δ cells to a similar extent as Pmk1^Sp^–HA (Figure 3C,D), but displayed a slightly lower activation threshold in response to an osmotic-saline stress (Figure 3E). Moreover, insertion of the N-lobe 24 aa motif within *S. japonicus* Pmk1 (Pmk1^Sj^ (ins82-105)–HA), resulted in a total lack of MAPK activity and functions (Figure 3B–E). Taking into account these findings, we hypothesized that the differences in biological activity among the different Pmk1 chimeras might result from changes in their binding affinity to the upstream activating MAPKK Pek1 [1,5]. Indeed, co-IP experiments revealed that the In Vivo association of Pmk1^Sj^–HA to a genomic Pek1–GFP fusion was enhanced as compared to that of Pmk1^Sp^–HA, and absent in cells expressing either Pmk1^Sp^(Δ82-105)–HA or Pmk1^Sj^-ins(82-105)–HA fusions (Figure 3F). Together, these observations indicate that Pmk1^Sj^ is fully functional in *S. pombe*, and that absence of the subdomain IV–V motif within the ATP-binding pocket at the N-terminal lobe is required for MAPKK binding and functional activation of the MAPK. They also suggest that the enhanced MAPKK binding and altered activity and activation pattern of Pmk1^Sj^ with regard to the *S. pombe* isoform (i.e. increased basal activity and reduced activation in response to stress), may rely upon structural constraints determined by its atypical N-terminal lobe.

**Figure 4:**
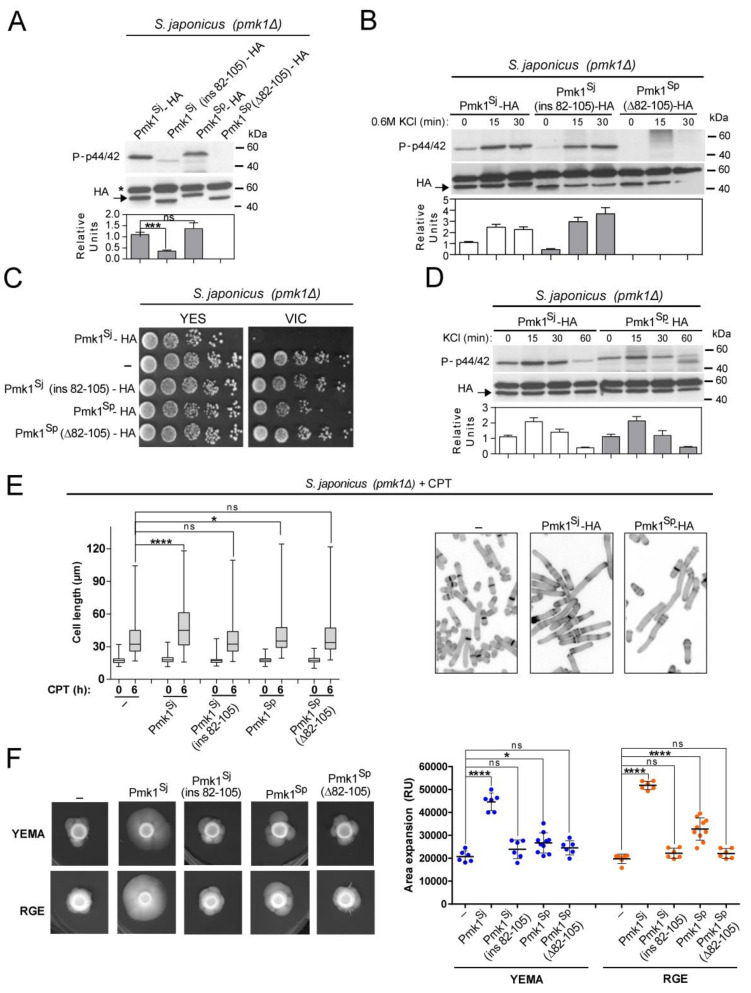
*S. pombe* Pmk1 shows little ability to execute CIP functions and to promote mycelial development in *S. japonicus.* (**A**) *S. japonicus pmk1*Δ cells expressing the Pmk1–HA chimeric constructs described in Figure 3A were grown in YES medium to mid-log phase, and activated/total Pmk1 were detected with anti-phospho-p44/42 and anti-HA antibodies, respectively. Relative units as mean ± SD (biological triplicates) for Pmk1 phosphorylation (anti-phospho-p44/42 blot) were determined with respect to the anti-HA blot (total Pmk1). ***, *p* < 0.001; ns, not significant, as calculated by unpaired Student’s *t*-test; (**B**) *S. japonicus pmk1*Δ strains expressing the Pmk1–HA chimeric constructs were grown in YES medium to mid-log phase and treated with 0.6 M KCl for the indicated times. Activated/total Pmk1 were detected with anti-phospho-p44/42 and anti-HA antibodies, respectively. Relative units as mean ± SD (biological duplicates) for Pmk1 phosphorylation (anti-phospho-p44/42 blot) were determined with respect to the anti-HA blot (total Pmk1); (**C**) Decimal dilutions of *S. japonicus pmk1*Δ cells (-), and those expressing Pmk1–HA chimeric constructs, were spotted onto YES plates supplemented with 0.5 µg/mL FK506 plus 0.2 M MgCl_2_ (VIC), and incubated for 3 days at 30 °C before being photographed. A representative experiment is shown; (**D**) *S. japonicus pmk1*Δ strains expressing the Pmk1–HA chimeric constructs were grown in YES medium to mid-log phase and treated with 0.6 M KCl for the indicated times. Activated/total Pmk1 were detected with anti-phospho-p44/42 and anti-HA antibodies, respectively. Relative units as mean ± SD (biological duplicates) for Pmk1 phosphorylation (anti-phospho-p44/42 blot) were determined with respect to the anti-HA blot (total Pmk1); (**E**) *Left*: Exponentially growing *S. japonicus pmk1*Δ cells (-), and those expressing the indicated Pmk1–HA chimeric constructs were inoculated at an initial cell density of 10^6^ cells/mL in YES medium with 6% glucose and incubated for 0 and 6 h with 0.2 μM CPT. Cell length at each time point is represented as box and whisker plots. Data obtained after quantification of one experiment performed per triplicate (*n* ≥ 400 cells/strain) are shown. *, *p* < 0.05; ****, *p* < 0.0001; ns, not significant, as calculated by one-way ANOVA. *Right*: representative images were obtained by fluorescence microscopy after calcofluor white staining; (**F**) *Left*: cells from log-phase cultures of *S. japonicus pmk1*Δ cells (-), and those expressing Pmk1–HA chimeric constructs growing in YES medium (~2.10^6^ total cells), were spotted on YEMA and RGE plates, incubated at 30 °C for 7 days, and then photographed. *Right*: the total area of mycelial expansion (expressed as relative units) was measured for each strain genotype (*n* ≥ 6) and is represented as a scatter plot. *, *p* < 0.05; ****, *p* < 0.0001; ns, not significant, as calculated by one-way ANOVA.

Insertion of the *S. pombe* 24 aa motif within *S. japonicus* Pmk1 (Pmk1^Sj^ (ins82-105)–HA), resulted in a clear reduction in basal MAPK activity as compared to the wild-type Pmk1^Sj^–HA fusion when expressed in a *S. japonicus pmk1*Δ background (Figure 4A). In contrary, MAPK activation during osmotic–saline stress remained unaffected (Figure 4B). However, Pmk1^Sj^(ins82-105)–HA did not abolish the VIC phenotype of *S. japonicus pmk1*Δ cells as the Pmk1^Sj^–HA version did (Figure 4C). Interestingly, a genomic Pmk1^Sp^–HA chimera expressed in *S. japonicus* showed a relative basal MAPK activity comparable to that of the endogenous Pmk1^Sj^–HA construct (Figure 4A), and became activated to a similar threshold in response to KCl stress (Figure 4D). Nonetheless, Pmk1^Sp^ was not fully functional in *S. japonicus*, because it suppressed the VIC phenotype of the *pmk1*Δ mutant only partially (Figure 4B). Finally, deletion of the Pmk1^Sp^ N-lobe motif (Pmk1^Sp^ (Δ82-105)–HA) resulted in a biologically inactive and non-functional MAPK chimera (Figure 4A–C).

As compared to endogenous Pmk1^Sj^–HA, expression of Pmk1^Sp^–HA showed a limited ability to promote filamentation in *S. japonicus pmk1*Δ cells during the initial steps of yeast to hypha differentiation induced with CPT (Figure 4E), and also at later stages, as confirmed by comparatively scoring the mycelial area of expansion of strains expressing both constructs growing on YEMA and RGE plates (Figure 4F). As might be expected, neither Pmk1^Sj^ (ins82-105)–HA nor Pmk1^Sp^ (Δ82-105)–HA fusions were able to alleviate the myceliation defect of the *pmk1*Δ mutant (Figure 4F). Therefore, these observations suggest that in *S. japonicus*, evolutionary divergence of MAPK Pmk1 secondary structure at the N-lobe has had a significant impact on its biological functions including the positive modulation of dimorphism.

## 4. Discussion

In this work, we have investigated the signaling architecture and biological functions of the cell integrity MAPK signaling pathway (CIP) in the dimorphic fission yeast *S. japonicus*. Comparative phenotypic assessment of *S. japonicus* and *S. pombe pmk1*Δ mutants revealed that control of ionic homeostasis and cell wall integrity is a conserved regulatory feature of the CIP in both fission yeast species. In contrast to *S. pombe*, Pmk1 did not localize to the septum in *S. japonicus*, and *pmk1*Δ cells only displayed cytokinetic defects in response to specific environmental cues (i.e. with KCl), suggesting that the CIP may not play a similar role during control of septation in both fission yeasts [1,34]. Pmk1-dependent regulation of cell separation in *S. pombe* operates in a manner mostly dependent on its localization at the division area; however, transcriptional control by nuclear Pmk1 has also been proposed to play some role during cytokinetic control [34]. Moreover, it has been recently shown that CIP/Pmk1 signaling participates in a checkpoint that relies on the SIN (septation initiation network), which ensures that *S. pombe* cytokinesis terminates successfully [35]. This regulatory mechanism could not have evolved in *S. japonicus*, since both fission species show marked differences in the timely coordination of mitosis and cytokinesis, with *S. japonicus* delaying the assembly of the CAR until late anaphase when chromosome segregation is complete [42]. Hence, it might be possible that Pmk1-dependent regulation of cytokinesis in *S. japonicus* is exerted at a transcriptional level.

*S. japonicus* Pmk1 becomes activated as with its *S. pombe* MAPK counterpart in response to environmental cues including saline and cell wall stress, and is negatively regulated by the SAPK pathway, as initially described in *S. pombe* [4]. However, unlike *S. pombe* Pmk1, which becomes strongly activated upon glucose withdrawal [3], Pmk1 activity undergoes a sudden decrease in *S. japonicus* cells after carbon source removal from the growth medium. Pmk1 activation in the absence of glucose is required in *S. pombe* for adaptive growth from fermentative to respiratory metabolism, and the use of alternative carbon sources such as glycerol [3]. Lack of Pmk1 activation in *S. japonicus* might thus be explained from an evolutionary perspective, since this fission yeast species is respiration defective, and utilizes glucose exclusively via fermentation [17].

The role of PKC orthologs Pck1 and Pck2 as key upstream regulators of the CIP pathway is maintained in *S. japonicus* as originally shown in *S. pombe* [1,6,7,39], although with several differences. As in *S. pombe*, *S. japonicus* Pck2 is the major activator of Pmk1 during unperturbed growth, whereas Pck1 deletion prompts increased Pmk1 phosphorylation. In *S. pombe*, this response has been shown to arise from a cell wall stress that is transduced to the CIP by Rho GTPases Rho1 and Rho2 [7], and whose existence in *S. japonicus* remains unknown. However, Pmk1 activation during cell wall damage, which in *S. pombe* relies on both Pck1 and Pck2, is in *S. japonicus* exclusively Pck2 dependent. These findings, together with the observation that *pck1*Δ cells are growth resistant to caspofungin, suggest that opposite control of CIP/Pmk1 activity by Pck1 and Pck2 has a meaningful impact on *S. japonicus* cell wall structure and integrity.

*S. japonicus* yeast cells readily differentiate to hyphae in response to different environmental cues [18,19,20,21]. This led us to thoroughly study the putative role of CIP signaling during regulation of the dimorphic transition in this organism. As proposed earlier [22], Pmk1 MAPK is an essential and positive modulator of both the early and late stages of *S. japonicus* yeast to hyphae differentiation. This role is similar to that played by cell integrity MAPKs during hyphal or pseudohyphal differentiation in other yeast species, including *Candida albicans* [43,44], *Cryptococcus neoformans* [45], and *Saccharomyces cerevisiae* [46]. However, our data also suggest that Pck1 and Pck2 may regulate CIP signaling during *S. japonicus* dimorphic switch in an intricate fashion. Deletion of Pck2, which strongly reduces Pmk1 basal activity, had a notoriously negative impact during the early steps of yeast to hyphae differentiation, and was very similar to that imposed by Pmk1 deletion. This suggests that the cellular requirement for basal CIP/Pmk1 signaling is maximal during the early stages of *S. japonicus* hyphal differentiation. On the contrary, Pck2 absence did not affect late mycelia expansion, indicating that only a minimal CIP activity threshold is necessary at the later stages of mycelia formation. On the other hand, Pck1 deletion, which increases basal Pmk1 activity, resulted both in increased hyphal elongation and myceliation during the whole differentiation process. Intriguingly, Pck1 negative regulation of *S. japonicus* yeast to hyphae switch is totally dependent on Pmk1 downregulation during late mycelial expansion, but appears to operate independently of the MAPK at the early stages of yeast differentiation. Altogether, these findings point to the existence of an antagonistic and biologically relevant control of *S. japonicus* hyphal differentiation by Pck1 and Pck2, which operates by fine-tuning of Pmk1 activity during the different stages of mycelia formation, but may also function independently of CIP signaling.

MAPKs adopt a typical kinase fold including N- and C-terminal lobes that mediate, respectively, ATP and effector/substrate binding, and are connected via a conserved hinge region at subdomain V [47]. Activation loop phosphorylation by the upstream MAPKKs results in MAPKs rotation of the N- and C-terminal lobes through this hinge region to facilitate phosphoryl transfer, and also triggers global changes in their conformational exchange dynamics [47]. Therefore, the atypical secondary structure of the N-lobe in *S. japonicus* Pmk1, which lacks a highly conserved motif corresponding to subdomain IV and part of the hinge region including the putative gatekeeper residue, raised the question of to what extent has evolutionary divergence modeled the biological functions of this MAPK? By performing kinase swapping experiments, we found that Pmk1^Sj^ is totally functional when expressed in *S. pombe*, as evidenced by its ability to become fully activated during growth and stress, and to suppress the altered ionic homeostasis and defective cytokinesis of *S. pombe pmk1*Δ cells. On the contrary, the ability of Pmk1^Sp^ to relieve the VIC phenotype and the hyphal differentiation defect of *S. japonicus pmk1*Δ cells was minimal. This behavior does not result from limited binding and activation of Pmk1^Sp^ by the MAPKK, since its basal activity and activation pattern in response to stress were very similar to those shown by Pmk1^Sj^. Thus, with its atypical structure, Pmk1^Sj^ seems more efficient for downstream substrate and/or effector docking than Pmk1^Sp^. Significantly, Pmk1^Sj^ conformational change, elicited by insertion of the highly conserved Pmk1^Sp^ N-lobe motif, resulted in a total lack of kinase function when expressed in *S. pombe* due to abrogated binding and activation by Pek1 MAPKK, but also in *S. japonicus*, despite becoming activated during growth and stress to a certain threshold. These results suggest that evolutionary divergence within the *Schizosaccharomyces* clade in the architecture of the Pmk1 N-terminal lobe has exerted a profound impact on MAPK binding/docking to both upstream (MAPKK) and downstream substrates/effectors. In support of this prediction, bioinformatics and structural studies with mammalian MAPKs have shown that co-evolution between the subdomains IV–V, the D-domain docking site, and the C-tail, play a major role for tight and allosteric regulation of MAPK activity [48].

In conclusion, while some of the functional roles of the CIP earlier described in *S. pombe* are conserved in *S. japonicus*, others have evolved separately in both fission yeast species. This includes the apparent inability of *S. japonicus* Pmk1 to become activated in response to glucose deprivation and the differential regulation of cytokinesis. Our findings also reveal an antagonistic role of PKC orthologs Pck1 and Pck2 in controlling MAPK basal activity during the regulation of yeast to hyphae dimorphic switch, and suggest that conformational changes in the N-lobe of *S. japonicus* Pmk1 have modeled CIP signaling to fulfill the specific developmental requirements of this fission yeast species.

## Data Availability

The data presented in this study are available on request from the corresponding authors.

## Supplementary Materials

The following are available online at https://www.mdpi.com/article/10.3390/jof7060482/s1; Figure S1: Septation phenotypes of *S. japonicus pmk1*Δ cells; Figure S2: Conserved structural features of Pmk1 MAPKs in *S. pombe* and *S. japonicus*; Figure S3: Conserved sequences surrounding the N-lobe subdomains IV and V in several fungal CIP MAPKs; Figure S4: Conserved structural features of Pck1 orthologs in *S. pombe* and *S. japonicus*; Figure S5: Conserved structural features of Pck2 orthologs in *S. pombe* and *S. japonicus*; Table S1: *S. japonicus* and *S. pombe* strains used in this work; Table S2: Oligonucleotides used in this work.
