# Supplementary material for: Specific Functional Features of the Cell Integrity MAP Kinase Pathway in the Dimorphic Fission Yeast Schizosaccharomyces japonicus
Source: J Fungi (Basel). 2021 Jun 14;7(6):482. doi: 10.3390/jof7060482 (PMC8232204; doi:10.3390/jof7060482)
Supplement: Supplementary file 1 [file jof-07-00482-s001.zip › jof-1245550-supplementary.pdf]

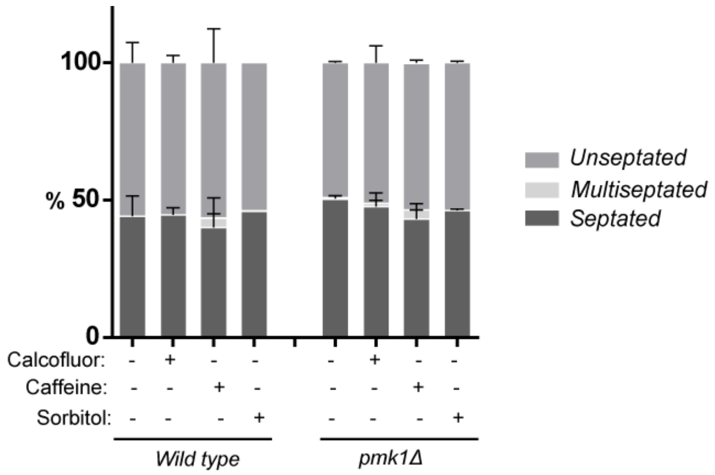

Figure S1. Septation phenotypes of *S. japonicus pmk1*Δ cells. Exponentially growing *S. japonicus* wild type and *pmk1*Δ strains were incubated for 10 h in YES medium plus 5 µg/ml calcofluor white, 5 mM Caffeine or 1M sorbitol, and the percentage of unseptated, septated and multiseptated cells (represented as mean $\pm$ SD from biological triplicates; number of total cells $\geq$200) was determined by fluorescence microscopy after calcofluor white staining.

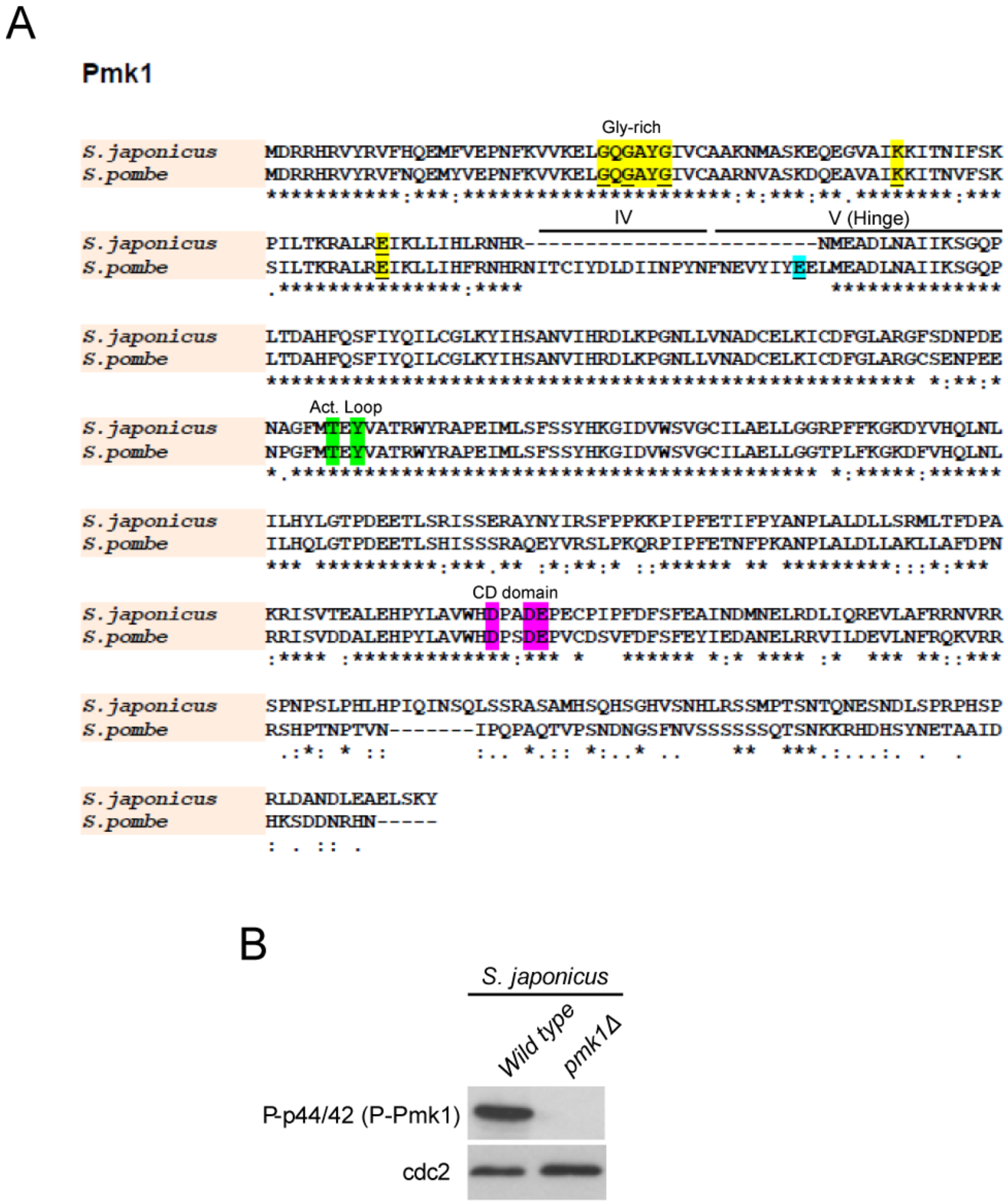

Figure S2. Conserved structural features of Pmk1 MAPKs in *S. pombe* and *S. japonicus*. (A) ClustalW analysis (https://www.genome.jp/tools-bin/clustalw) of amino-acids sequences of Pmk1 MAPKs in *S. pombe* and *S. japonicus*. '*': identical amino acid; ':': conserved substitution, '.': semi-conserved substitution. Conserved residues/motifs involved in ATP binding are shaded in yellow. The gate-keeper residue is shaded in blue. Putative common docking (CD) sites are shaded in purple. The phosphorylatable amino acids at the conserved -TEY- activation loop are shaded in green. (B) *S. japonicus* wild type and *pmk1*Δ strains were grown in YES medium to mid-log phase, and activated Pmk1 was detected with anti-phospho-p44/42 antibody, whereas anti-Cdc2 was used as a loading control.

```
Chaetothyriales DV737_g786        MSDLSGRKVFKVFNQDFIVDERYTVTKELGQGAYGIVCAATNTNTG--EG
Chaetothyriales DV736_g4926       MSDLSGRKVFKVFNQDFIVDDRYTVTKELGQGAYGIVCAATNTQTG--EG
O. camponoti CP532_1836           MAGLQGRKVFKVFNQDFIVDERYTVTKELGQGAYGIVCAAVNNQTT--EG
S. japonicus Pmk1                 --MDRRHRVYRVFHQEMFVEPNFKVVKELGQGAYGIVCAAKNMASKEQEG
S. pombe Pmk1                     --MDRRHRVYRVFNQEMYVEPNFKVVKELGQGAYGIVCAARNVASKDQEA
                                        ::*::**:*:: *: .:.*.************** *  :   *.
                                                                           IV
Chaetothyriales DV737_g786        VAIKKVTNVFSKKILAKRALREIKLLQHFRGHRNV---------------
Chaetothyriales DV736_g4926       VAIKKVTNVFSKKILAKRALREIKLLQHFRGHR-----------------
O. camponoti CP532_1836           VAIKKVTNIFSKKILAKRALREIKLMQHFRGHR-----------------
S. japonicus Pmk1                 VAIKKITNIFSKPILTKRALREIKLLIHLRNHR-----------------
S. pombe Pmk1                     VAIKKITNVFSKSILTKRALREIKLLIHFRNHRNITCIYDLDIINPYNFN
                                  *****:**:*** **:*********: *:*.**
                                           V (Hinge)
Chaetothyriales DV737_g786        ------ELMECDLAAIIRSGQPLTDAHFQSFIYQILCGLKYIHSANVLHR
Chaetothyriales DV736_g4926       ------NLMECDLAAIIRSGQPLTDAHFQSFIYQILCGLKYIHSANVLHR
O. camponoti CP532_1836           ------NLMECDLAAIIRSGQALTDAHFQSFIYQILCGLKYIHSANVLHR
S. japonicus Pmk1                 -------NMEADLNAIIKSGQPLTDAHFQSFIYQILCGLKYIHSANVIHR
S. pombe Pmk1                     EVYIYEELMEADLNAIIKSGQPLTDAHFQSFIYQILCGLKYIHSANVIHR
                                          **.** ***:***.*************************:**
```

Figure S3. Conserved sequences surrounding the N-lobe subdomains IV and V in several fungal CIP MAPKs. MAPKs ClustalW analysis (https://www.genome.jp/tools-bin/clustalw) of sequences surrounding the N-lobe subdomains IV and V in of Pmk1 MAPKs in *S. pombe* and *S. japonicus*, and putative MAPKs from *Chaetothyriales* (Gen Pep: DV737_g786 and DV736_g4926), and *Ophiocordyceps camponoti* (Gen Pep: CP532_1836). '*': identical amino acid; ':': conserved substitution, '.': semi-conserved substitution.

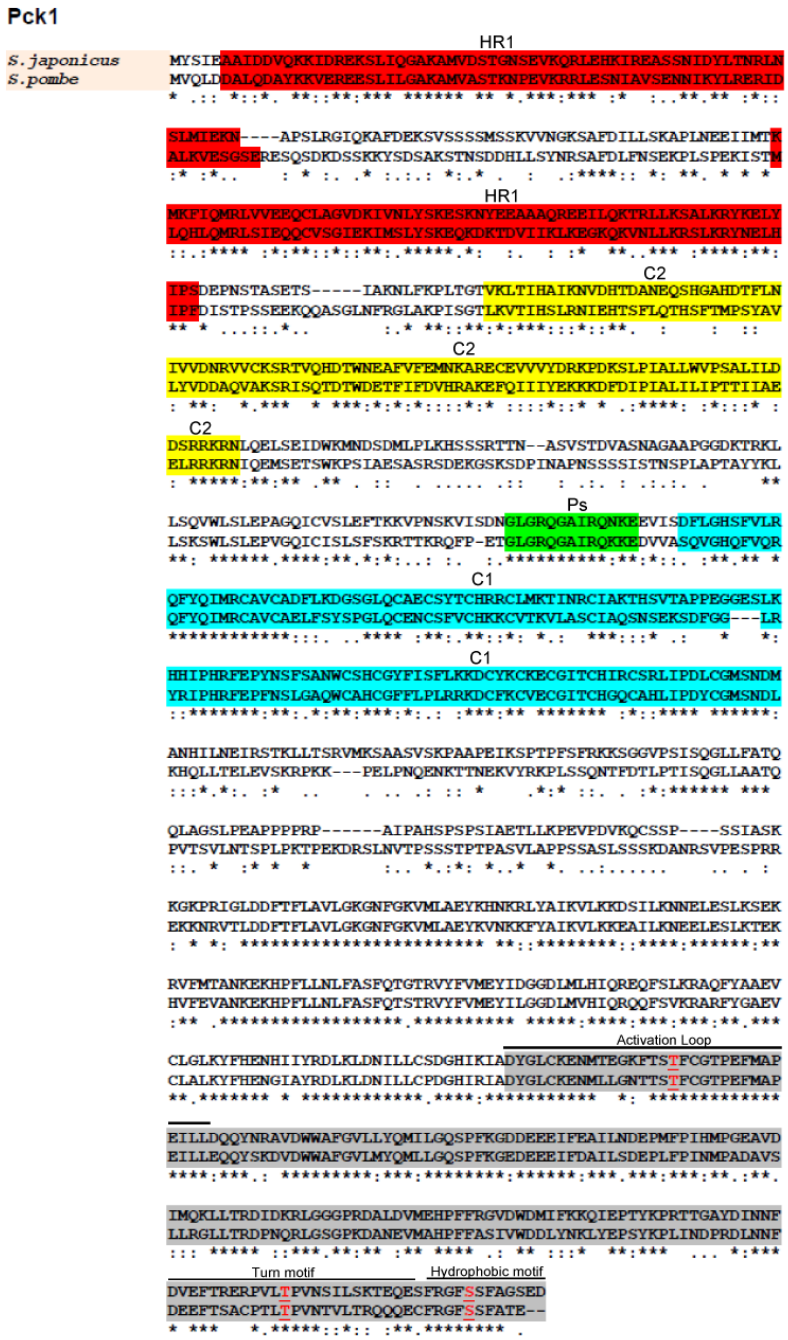

Figure S4. Conserved structural features of Pck1 orthologs in *S. pombe* and *S. japonicus*. Conserved domain structure of Pck1 orthologs from *S. pombe and S. japonicus* obtained after protein sequence alignment by CLUSTALW (https://www.genome.jp/tools-bin/clustalw). '*': identical amino acid; ':': conserved substitution, '.': semi-conserved substitution. Labels: HR1 (red), putative rho-binding repeat; C2 (yellow), putative Ca(2+)-binding motif; Ps (green), putative pseudosubstrate motif; C1 (blue), putative diacylglycerol binding motif. Amino acid sequence alignment of Pck1 and Pck2 is also shown. Conserved canonical phosphorylated residues at the activation loop (AL), turn motif (TM), and hydrophobic motif (HM) within the catalytic domain (gray) are marked in red.

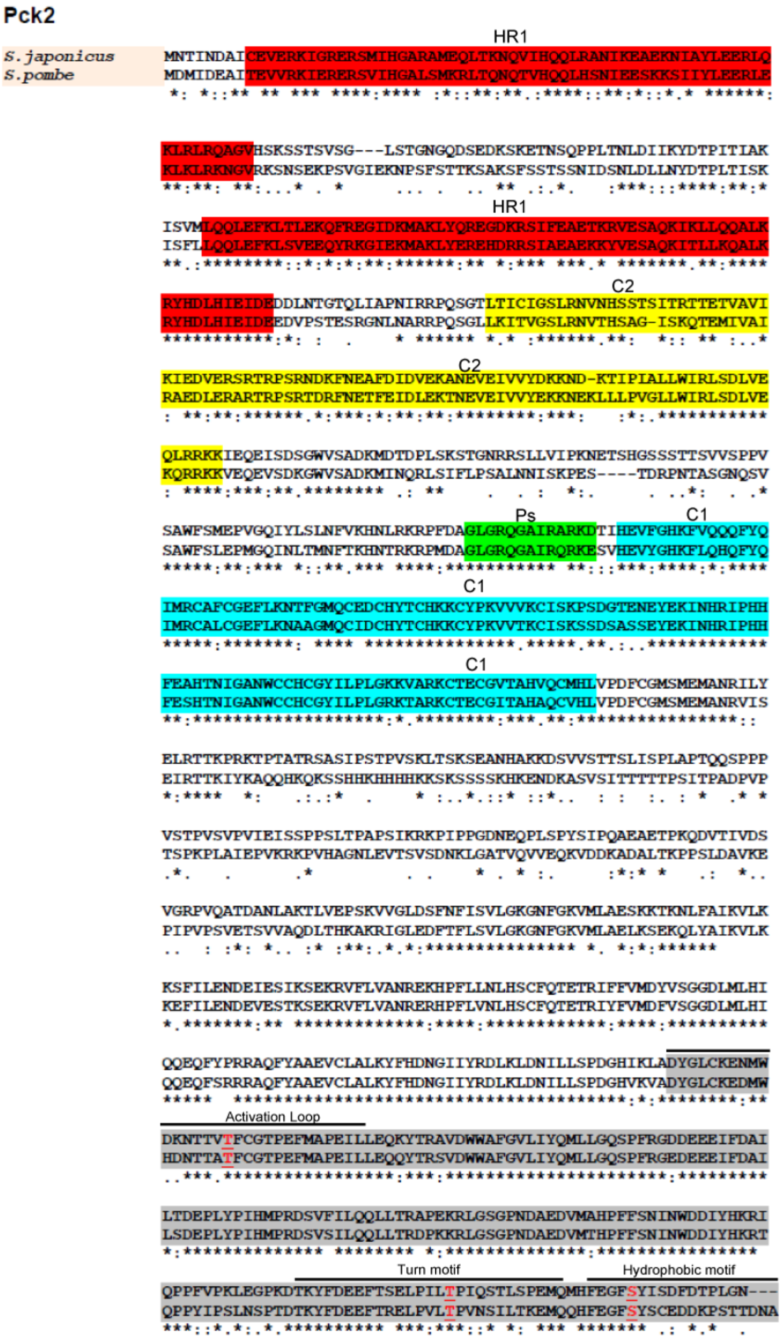

Figure S5. Conserved structural features of Pck2 orthologs in *S. pombe* and *S. japonicus*. Conserved domain structure of Pck2 orthologs from *S. pombe and S. japonicus* obtained after protein sequence alignment by CLUSTALW (https://www.genome.jp/tools-bin/clustalw). '*': identical amino acid; ':': conserved substitution, '.': semi-conserved substitution. Labels: HR1 (red), putative rho-binding repeat; C2 (yellow), putative Ca(2+)-binding motif; Ps (green), putative pseudosubstrate motif; C1 (blue), putative diacylglycerol binding motif. Amino acid sequence alignment of Pck1 and Pck2 is also shown. Conserved canonical phosphorylated residues at the activation loop (AL), turn motif (TM), and hydrophobic motif (HM) within the catalytic domain (gray) are marked in red.

Table S1. ***S. japonicus*** **and** ***S. pombe*** **strains used in this work.**

S. japonicus strains	**Genotype**	**Source**
NIG2028	*h*$^{-}$ *prototroph*	Furuya & Niki 2009
NIG5091	*h*$^{-}$ *ura4-D3*	Furuya & Niki 2009
EGJ4	*h*$^{-}$ *pmk1::ura4*$^{+}$ *ura4-D3*	This work
EGJ108	*h*$^{?}$ *pck1::NatMX6*	This work
EGJ73	*h*$^{-}$ *pck2::ura4*$^{+}$ *ura4-D3*	This work
EGJ128	*h*$^{?}$ *pck1::NatMX6 pmk1::ura4*$^{+}$	This work
EGJ83	*h*$^{-}$ *pmk1::NatMX6 ura4-D3*	This work
PH6	*h*$^{-}$ *pmk1*^{Sj}*-GFP::NatMX6*	This work
EGJ140	*h*$^{-}$ *sty1::ura4*$^{+}$ *pmk1*^{Sj}*-GFP::NatMX6*	This work
EGJ99	*h*$^{-}$ *pmk1*^{Sj}*-HA::ura4*$^{+Sj}$ *pmk1::NatMX6 ura4-D3*	This work
EGJ132	*h*$^{-}$ *pmk1*^{Sj}*(ins 82-105)-HA::ura4*$^{+Sj}$ *pmk1::NatMX6 ura4-D3*	This work
EGJ96	*h*$^{-}$ *pmk1*^{Sp}*-HA::ura4*$^{+Sj}$ *pmk1::NatMX6 ura4-D3*	This work
EGJ102	*h*$^{-}$ *pmk1*^{Sp}*(Δ 82-105)-HA::ura4*$^{+Sj}$ *pmk1::NatMX6 ura4-D3*	This work
S. pombe strains	**Genotype**	**Source**
MM1	*h*$^{+}$	Lab stock
MI102	*h*$^{+}$ *pmk1::KanMX6*	Madrid *et al*. (2007)
MM1904	*h*$^{-}$ *pmk1::NatMX6*	Madrid *et al*. (2017)
BV829	*h- pck1::ura4*$^{+}$	Dr Takashi Toda
MM1197	*h*$^{?}$ *pck2::NatMX6*	This work
LS111	*h*$^{+}$ *pmk1*^{Sp}*-GFP:leu1*$^{+}$ *pmk1::KanMX6*	Sánchez-Mir *et al.* (2012)
EG685	*h*$^{-}$ *pmk1*^{Sj}*-HA:leu1*$^{+}$ *pmk1::NatMX6*	This work
EG686	*h*$^{-}$ *pmk1*^{Sj}*(ins 82-105)-HA: leu1*$^{+}$ *pmk1::NatMX6*	This work
EG682	*h*$^{-}$ *pmk1*^{Sp}*-HA: leu1*$^{+}$ *pmk1::NatMX6*	This work
EG684	*h*$^{-}$ *pmk1*^{Sp}*(Δ 82-105)-HA: leu1*$^{+}$ *pmk1::NatMX6*	This work

All *S. pombe* strains are *ade6-M216 leu1-32 ura4D-18.*

Table S2. Oligonucleotides used in this work.

OLIGONUCLEOTIDE	SEQUENCE 5'-3'	Use
Pmk1DJp-W	CCGCGTTTAATTACGATCATTCACC	*pmk1*$^{+}$ deletion
Pmk1DJp-X (Ura4)	GAGCGGAAGAACGGAATCGTGGCGGCCGATGGTATTCCTTGCACT CGAGAC	*pmk1*$^{+}$ deletion (*Ura4*$^{+}$)
Pmk1DJp-Y (Ura4)	GCAGTGCGGTATCGTATAATTAGTGTCTAAGCAAGATACTGAAAGC TGGC	*pmk1*$^{+}$ deletion (*Ura4*$^{+}$)
Pmk1DJp-X (NatR)	GGGGATCCGTCGACCTGCAGCGTACGAGATGGTATTCCTTGCAC TCGAGAC	*pmk1*$^{+}$ deletion (*NatR*)
Pmk1DJp-Y (NatR)	GTTTAAACGAGCTCGAATTCATCGATCTAAGCAAGATACTGAAAG CTGGC	*pmk1*$^{+}$ deletion (*NatR*)
Pmk1DJp-Z	ACTACCACTTCTCACTACATTTCTG	*pmk1*$^{+}$ deletion
Pmk1DJp-Comp2-5'	CGCGTTCACTCACATTTCTTCGTG	Confirmation of *pmk1*$^{+}$ deletion.
Pck1DJp-W	CACGCAGTTCTGTGTATTCGGTTTT	*pck1*$^{+}$ deletion
Pck1DJp-X (NatR)	GCGCGCCTTAATTAACCCGGGGATCCGTCGCGCGACTAAATTTAGC AATGCTTAG	*pck1*$^{+}$ deletion (*NatR*)
Pck1DJp-Y (NatR)	CCATCCAGTTTAAACGAGCTCGAATTCATCGTGTTCCTCGTGCGCG CTTACGAACA	*pck1*$^{+}$ deletion (*NatR*)
Pck1DJp-Z	AGCAGCGAATTAAAGCGTCAATTCC	*pck1*$^{+}$ deletion
Pck1DJp-Comp-5'	TTCTTCAACTGGAGGATTCTGTAAC	Confirmation of *pck1*$^{+}$ deletion.
NatR-Comp-R	TTATTGTCAGTACTGATTAGGGGCA	Confirmation of *NatR* deletions.
Pck2DJp-W	CTTCGTATGCTCCATTCTTGATTGT	*pck2*$^{+}$ deletion
Pck2DJp-X (Ura4)	GAGCGGAAGAACGGAATCGTGGCGGCCTTCTTTAAGGGAAACCGT GAGGTAC	*pck2*$^{+}$ deletion (*Ura4*$^{+}$)
Pck2DJp-Y (Ura4)	GCAGTGCGGTATCGTATAATTAGTGTCCTCATTAAGCATTTCACGG CGATC	*pck2*$^{+}$ deletion (*Ura4*$^{+}$)
Pck2DJp-Z	GTTCTACGGTCTTGATCTGCTGAAC	*pck2*$^{+}$ deletion
Pck2DJp-Comp-5'	TTCTTCAACTGGAGGATTCTGTAAC	Confirmation of *pck2*$^{+}$ deletion.
Ura4Sj-Comp-R	CTTCTTGGCGACTGCATTAGGATGC	Confirmation of *Ura4*$^{+}$ deletion.
Pmk1Sj:GFP-*Sal*I-5´	ATAATGTCGACGTATGGCAACCATGTGGTAGAC	*pmk1*$^{+}$ C-terminal tagging
Pmk1Sj:GFP-*Pac*I-3´	ATAATGTTAATTAAGTACTTGGACAGTTCTGCCTC	*pmk1*$^{+}$ C-terminal tagging
GFP-Comp-5'	CTGCCCTTTCCAAAGATCCCAACG	Confirmation of C-terminal tagging
Sty1Djp-W	GCATGGGCGCGTTCGGTCTTGTATG	*sty1*$^{+}$ deletion
Sty1Djp-X (URA4)	GAGCGGAAGAACGGAATCGTGGCGGCCACACCAGCACTGTGGACGT ACTTC	*sty1*+ deletion (*Ura4*$^{+}$)
Sty1Djp-Y (URA4)	GCAGTGCGGTATCGTATAATTAGTGTGACGATCTGCAGCAAGAATAC ATTG	*sty1*+ deletion (*Ura4*$^{+}$)
Sty1Djp-Z	TGCTTGCACCTCACTATCCACATTATG	*sty1*$^{+}$ deletion
Sty1jp-COMP5'	ATGGCTGAATTTGTTCGTACACAGAT	Confirmation of *sty1*$^{+}$ deletion.
PromPmk1Jp-*Not*I-5´	ACTTAGCGGCCGCCCGCGTTTAATTACGATCATTCACC	*pmk1*$^{+Sj}$ ORF Cloning and C-terminal tagging
Pmk1Jp:HA-*BamH*I-3´	ACTTAGGATCCTTAGCCCGCATAGTCAGGAACATCGTATGGGTAGC CGTACTTGGACAGTTCTGCCTCGAGATC	*pmk1*$^{+Sj}$ ORF Cloning and C-terminal tagging
Pmk1Jp-Exon-5´	ACTTGCATTTACGATCTCGACATTATAAATCCATACAATTTTAATGAA GTTTACATTTACGAAGAGATGGAAGCAGATTTAAATGCCATT	*pmk1*$^{+Sj}$ ORF Cloning and *pmk1*$^{+Sp}$ Exon2 insertion
Pmk1Jp-Exon-3´	AATGTCGAGATCGTAAATGCAAGTAATATTCCTGTGATTCCGAAGAT GAATC	*pmk1*$^{+Sj}$ ORF Cloning and *pmk1*$^{+Sp}$ Exon2 insertion
PromPmk1Sp-*Xba*I-5´	ACTTATCTAGATTTCTCATTGCCGCTTCTG	*pmk1*$^{+Sp}$ ORF Cloning and C-terminal tagging
Pmk1Sp-HA-*Sma*I-3´	CCTTACCCGGGTTAGTGGTGATGATGGTGATGTGCATAGTCCGG GACGTCATAGGGATAGCCCGCATAGTCAGGAACATCGTATGGGT AGGATCCGTTATGGCGATTATCATCAGAC	*pmk1*$^{+Sp}$ ORF Cloning and C-terminal tagging
Pmk1Sp-Δ Exon-5´	CATTTTCGTAATCATCGTAACATGGAGGCCGATTTGAATG	*pmk1*$^{+Sp}$ ORF Cloning and Exon2 deletion.
Pmk1Sp-Δ Exon-3´	CATTCAAATCGGCCTCCATGTTACGATGATTACGAAAATG	*pmk1*$^{+Sp}$ ORF Cloning and Exon2 deletion.
Pck2-Del-F	CACATTAATATTAAAAACGGAAGCGAAAAATTGCAGATAAGAAGTTA AGCGGAAAAATCAGGATTAGGCCATTTAAAGTACGGATCCCCGGG TTAATTAA	*pck2*$^{+}$ deletion (*NatR*)
Pck2-Del-R	AAAAAGTCGAAATTAGAATAATTTATCAATGCAATGAAAGATTAAGA AAATGAGAGTAACTTTATGCTCAATTTAAGGTGGAATTCGAGCTCG TTTAAAC	*pck2*$^{+}$ deletion (*NatR*)
